# Awareness of Necessity to Call 9-1-1 for Stroke Symptoms, Upstate New York

**Published:** 2008-03-15

**Authors:** Janine M Jurkowski, Dayna M Maniccia, Barbara A Dennison, Steven J Samuels, Deborah A Spicer

**Affiliations:** Department of Health Policy, Management, and Behavior, University at Albany School of Public Health; Department of Health Policy Management and Behavior, University at Albany, School of Public Health, Rensselaer, New York; Bureau of Health Risk Reduction, Division of Chronic Disease Prevention and Adult Health, New York State Department of Health, Menands, New York; School of Public Health, Department of Epidemiology and Biostatistics, University at Albany, Rensselaer, New York; Healthy Heart Program, Bureau of Health Risk Reduction, New York State Department of Health, Albany, New York

## Abstract

**Introduction:**

Stroke is the third leading cause of death and a leading cause of disability in New York State. A New York study determined that only 19.9% of patients arrived at a designated stroke center within 3 hours of symptom onset. Yet, receiving treatment within 90 minutes of stroke symptom onset is optimal for improved outcomes. Delay in recognition of stroke symptoms and their severity contributes to treatment delay.

**Methods:**

A random-digit–dialed, list-assisted telephone survey about stroke knowledge was administered to 1789 adults aged 30 years or older in upstate New York in 2006. Bivariate and regression analysis were used to examine factors associated with intent to call 9-1-1 for symptoms of stroke.

**Results:**

The largest proportion of respondents (72.4%; 95% confidence interval [CI], 69.9%–74.8%) reported they would call 9-1-1 if they noticed they or someone else had difficulty speaking, and the fewest (33.3%; 95% CI, 30.7%–36.0%) respondents reported they would call 9-1-1 for trouble seeing or double vision. Multivariate analysis found that those who had a history of delay in getting medical care in the past 6 months had decreased odds of intending to call 9-1-1 for stroke symptoms (difficulty speaking: adjusted odds ratio [AOR], 0.76; 95% CI, 0.58–1.00; trouble seeing: AOR, 0.69; 95% CI, 0.53–0.91; facial droop: AOR, 0.85; 95% CI, 0.65–1.11; arm weakness: AOR, 0.80; 95% CI, 0.63–1.03). Age, education, and history of a stroke or heart event were not consistently associated with intent to call 9-1-1.

**Conclusion:**

Survey respondents do not interpret some stroke symptoms as urgent enough to activate the emergency medical system. History of delaying care is a behavioral pattern that influenced intent to call 9-1-1.

## Introduction

In the United States, stroke is the primary cause of severe long-term disability and the third leading cause of death (1). Stroke is also the third leading cause of death and a major cause of disability in New York State (2). In 2003, there were approximately 57,500 hospitalizations attributable to new and recurrent strokes in New York state (2). Although 50% to 70% of stroke survivors regain functional independence, 15% to 30% are permanently disabled, and 20% require institutional care after 3 months (3). Treatment of acute ischemic stroke with tissue-type plasminogen activator within 3 hours of symptom onset is recommended (4). If treatment for stroke is received early, the chance of recovery is improved (5). Ischemic stroke patients treated within 90 minutes of onset of symptoms have the most improvement (6).

A New York State pilot study was designed to determine whether designation of certain health care facilities as stroke centers and transport of stroke patients to these centers would improve care. The finding in that study was that 19.9% of patients arrived at a designated stroke center within 3 hours after the onset of symptoms (7). New York State's findings are similar to other studies, one of which showed that the median prehospital delay time was 2.6 hours, with approximately half of the patients delaying more than 3 hours (8). The delay in time until hospital arrival is a major obstacle for improving outcomes by obtaining treatment within 3 hours of symptom onset (9,10). Factors that contribute to the delay between symptom onset and hospital arrival include identification of stroke symptoms, determination that the symptoms require immediate emergency care, calling 9-1-1, and the time it takes from a 9-1-1 telephone call until hospital arrival (11).

Emergency medical services (EMS) response has not been found to be a major contributor to delay in hospital arrival. Once a call to 9-1-1 is made, it is estimated that the combined mean time of call to EMS arrival, on-scene time, and transport time is approximately 34 minutes (5). Studies show that patients who arrive by ambulance (34%–71% of stroke patients) (7,12-15) arrive sooner after onset of stroke symptoms than do patients who do not use EMS (15). This evidence suggests that most of the delay occurs before the call to 9-1-1 is made.

Research on knowledge of symptoms varies, probably because of methodological differences, including different ways of measuring symptom awareness and intent to call 9-1-1. When respondents were asked to select stroke signs or symptoms from a list, the percentage of people who were able to correctly identify symptoms (16-18) was much higher than when respondents were asked without prompting (19,20). A multistate study using data from the Behavioral Risk Factor Surveillance System (BRFSS) asked respondents to identify which symptoms were stroke symptoms. This study found that awareness of stroke symptoms varied by state, but overall awareness of individual symptoms when prompted was high: 94% for weakness of face, arm, or leg to 61.3% for sudden severe headache (17). In another study, loss of vision was the symptom least often identified, and sudden numbness was the most frequently identified symptom (16). A study that asked open-ended questions without prompting reported a lower percentage of respondents (57%) were able to list at least one of the symptoms (19). Ability to identify all symptoms was low, from 17% (17) to 46% (21) when prompted.

Research that asked respondents what they would do if they thought they were having a stroke suggests that awareness of the need for emergency care for a stroke is high, ranging from 70% (20) to 83% (16). However, when respondents were asked if they would call 9-1-1 when they had a symptom (not specifically said to be a stroke symptom), the percentages were lower (22). Participants reported that they would call 9-1-1 for 34.1% of the stroke symptoms identified. The rest would call a doctor or wait 1 hour or 1 day (22). The multistate BRFSS study found that, when asked to identify stroke symptoms from a list of symptoms, only 17.2% of respondents were aware of all five stroke symptoms *and* knew to call 9-1-1 for medical assistance when they thought someone was having a stroke or heart attack (17,23). Demographic factors associated with intent to call 9-1-1 are female sex, high income and education levels, and younger ages (21). The findings from these studies suggest that most people would call 9-1-1 if they knew they were or someone else was having a stroke. People recognize stroke as needing immediate care (20). However, the research indicates that people do not recognize the five symptoms of stroke (trouble seeing, facial droop, arm weakness, dizziness or loss of balance, and inability to speak or slurred speech). Therefore, people do not recognize the immediate need to call 9-1-1 for symptoms of stroke (17–20).

The purpose of this paper is to examine factors that are associated with respondents' intent to call 9-1-1 for signs and symptoms of stroke that occur in themselves or another person. The overall goal is to provide a better understanding of the reasons that result in patients not arriving at a hospital emergency department soon enough to receive the greatest benefit from treatment.

## Methods

### Setting

This paper presents baseline data from an intervention study designed to raise awareness of stroke signs and symptoms and the need to call 9-1-1. The baseline data are from adults aged 30 or older living in four upstate New York counties. In 2006, the four counties had 354,878 households with a total population of 979,680 persons, 13% of whom were older than 65 years. In 2005, the population was 81% non-Hispanic white, 9% black, 6% Hispanic, and 5% other. In 2000, 84% of people older than 25 years were high school graduates and 26% had at least a bachelor's degree (24). Between 1991 and 1998, the average rate of stroke mortality in the four counties was 101 per 100,000 persons; the rate was 89 per 100,000 for the entire state (25).

### Survey instrument

The survey instrument, consisting of 37 primarily close-ended questions, was developed to evaluate a stroke awareness media campaign. Information was collected on demographic characteristics, health care use, familiarity with EMS, and intended behavior in response to four stroke warning symptoms in oneself or another. The survey was administered in English.

Demographic and health history questions were based on the National Health Interview Survey (NHIS) (26), the National Health and Nutrition Examination Survey (NHANES) (27), the BRFSS survey (28), the Stroke Factor Survey (29) (A.T. Schneider, MD, oral and written communication, February 2006), and the Stroke Action Test (22). Respondents were asked what they would do if they noticed or were informed of one of the following signs and symptoms in themselves or another: an inability to speak correctly, slurred speech or use of inappropriate words, sudden trouble seeing or double vision, sudden arm weakness or a tingling sensation and numbness in the arm, stomach cramps, a temperature of 101 degrees F or higher for 12 hours or more, and sharp back pain between the shoulder blades (Appendix). Respondents were also asked what they would do if they noticed another person whose face looked uneven or drooped on one side or noticed another person who could not smile evenly. They were also asked what they would do if they experienced pain or burning during urination. Respondents were asked to answer by selecting one of the following options: 1) wait, watch symptoms, and then decide what to do; 2) call a family member or friend; 3) call a doctor or nurse; 4) call 9-1-1 or EMS. A history of delaying care was determined by respondents' answering whether they themselves delayed getting care for any reason (a list was provided) in the past 6 months. If the respondent answered yes to any reason listed or provided a reason that was not listed, he or she was considered to have delayed care in the past. Respondents were also asked if they or a family member had ever been told by a doctor that they had hypertension or high blood pressure or had had a heart attack or stroke.

### Sample population

Stratified list-assisted random-digit–dialing was used to select households for participation in the survey (30). The sampling frame consisted of all working telephone number banks with prefixes in the four upstate New York counties. Sampling was proportionate to the 2005 population of each county. Up to 15 attempts were made to reach a respondent. Once a household was reached, an adult aged 30 years or older was randomly selected to participate in the survey.

A total of 25,410 telephone numbers were generated for sampling. Of these, 9622 (38%) were nonworking or disconnected, 3846 (15%) were nonresidential, and 1098 (4%) were ineligible because they were out of the area, a fax or data line, or a cell phone. One hundred sixty-seven (<1%) households had no eligible respondent. Of the 10,677 numbers that were eligible for inclusion in the survey, 1789 (17%) interviews were completed. Only 6% of eligible respondents refused to participate in the survey. The final survey response rate, calculated using the American Association for Public Opinion Research Response Rate 3 method (31), was 36%.

### Data collection

The study protocol was approved by the University at Albany Institutional Review Board. The survey was administered by a professional survey research firm using procedures based on the BRFSS protocol (30). Calls were made during various times of the weekday and weekend. The survey was pilot tested before the beginning of data collection and administered from July through September of 2006. On average, respondents took 11.4 minutes to answer all survey questions.

### Statistical analyses

Variables previously shown to be associated with knowledge or intent to call 9-1-1 in response to stroke warning symptoms were included in the models as independent variables (3,5,10-12,16,18-21,23,29,32,33): education, age, sex, race, and personal and family history of heart attack and stroke. History of delaying receipt of medical care was also included. Reported intentions to call 9-1-1 in response to a given stroke sign or symptom in oneself or another were similar. Most respondents said they would do the same thing for a sign or symptom in themselves or another, and intention to call 9-1-1 for a sign or symptom in oneself and another was associated with similar demographic variables; therefore they were combined.

Age was collapsed into two groups: 30 to 64 and 65 or older. Race was classified into two groups: white alone, where white was the only race chosen by the respondent, and all others. No further racial/ethnic breakdown was possible because of the small sample of racial/ethnic groups other than white. Respondents were classified into two groups depending on their response to the sign or symptom questions. Respondents who answered they intended to call 9-1-1 in response to a stroke sign or symptom in themselves or another constituted the correct response group, while the referent group consisted of respondents who reported another response.

All analyses were conducted using Stata Version 9 (StataCorp, College Station, Texas). Frequency distributions of all variables were examined, and skip-pattern accuracy was verified. Frequency distributions reported are unweighted. The survey data were weighted in bivariate and multivariate analysis to reflect the probability of being selected and to more accurately represent the age, sex, and racial distribution of the population. The weights were computed to match the age, sex, and racial distributions for the combined area, not for the individual counties. The post-stratification factor was computed by the method of ratio raking (34).

Design-based *F* tests, uncorrected for multiple hypotheses testing, were conducted to assess the presence of binary relationships between predictor and outcome variables. Multivariate logistic regression, using the weighted population data, was used to identify factors associated with intention to call 9-1-1 in response to each of four stroke symptoms while controlling for demographic characteristics and event history.

## Results

### Sample description

The sample (n = 1789) was primarily female (65%), white (88%), and highly educated (68% had a college degree or college-level education; 20% had at least some graduate-level education). Most were aged 45 or older, and 25% were aged 65 or older. Almost all respondents (96%) had some form of health care coverage. Even though most respondents had health care coverage, 32% reported that they had delayed getting health care at least once in the past 6 months. Thirty-seven percent of respondents reported that they or a family member have been told by a doctor that they have had a heart attack or stroke. Sixty-two percent of respondents reported that they or a family member have been told by a doctor that they have or have had hypertension (Table 1).

### Knowledge of severity of stroke signs or symptoms

The largest proportion of respondents (72.4%, 95% confidence interval [CI], 69.9%–74.8%) reported that they would call 9-1-1 if they noticed an inability to speak in themselves or another. Approximately 33% reported that they would call 9-1-1 for trouble seeing or double vision, 64% for facial droop, and approximately 50% for arm weakness (Table 2). Among respondents delaying receiving medical care in the past 6 months, between 28.7% and 68.6% reported that they would call 9-1-1 in response to stroke signs or symptoms. Between 30.7% and 72.5% of women reported intention to call 9-1-1 in response to a stroke sign or symptom. For both age groups, fewer respondents reported intent to call 9-1-1 in response to trouble seeing or double vision (33.2%–33.8%) compared with the other signs or symptoms. For all demographic characteristics and health-related variables, intention to call 9-1-1 for inability to speak correctly was reported most frequently, and for trouble seeing or double vision was reported the least frequently.

A significant bivariate relationship existed between sex and intention to call 9-1-1 for trouble seeing or double vision; a larger proportion of men reported intention to call. Intention to call 9-1-1 for facial droop in another was related to age of less than 65. Calling 9-1-1 for having trouble seeing and arm weakness was related to respondents having no prior history of heart attack or a stroke in themselves or a family member. In contrast, a history of hypertension was associated with intention to call 9-1-1 for noticing an inability to speak correctly in oneself or another. People who reported delaying medical care in the past 6 months reported intention to call 9-1-1 less frequently for all four signs or symptoms (Table 2).

### Multivariate results

After controlling for other factors, the relationship between delayed medical care and intention to call 9-1-1 remained for all factors and was significant for inability to speak correctly (adjusted odds ratio [AOR], 0.76; 95% CI, 0.58–1.00) and trouble seeing (AOR, 0.69; 95% CI, 0.53–0.91). See Table 3 for the multivariate model findings.

Older age was significantly associated with decreased likelihood of intention to call 9-1-1 for facial droop (AOR, 0.67; 95% CI, 0.51–0.89). Compared with individuals who had not previously been told that they or a family member had had a heart attack or stroke, those who had been told were less likely to report intention to call 9-1-1 in response to trouble seeing or double vision (AOR, 0.75; 95% CI, 0.58–0.98). Having a graduate degree was significantly associated with increased likelihood of calling 9-1-1 in response to arm weakness (AOR, 1.51; 95% CI, 1.09–2.10) compared with having at most a high school education or General Educational Development certificate (Table 3).

## Discussion

The percentage of survey respondents from the four upstate New York counties who reported they would call 9-1-1 for a stroke symptom they identified in themselves or another varied depending on the symptom. The largest proportion of respondents reported that they would call 9-1-1 if they noticed an inability to speak correctly in themselves or someone else. The smallest proportion of respondents reported that they would call 9-1-1 if they or someone else experienced trouble seeing or double vision. One of the most striking observations is that those who delayed getting medical care in the past 6 months were less likely to call 9-1-1 for inability to speak correctly or trouble seeing or double vision than those who had not delayed getting medical care. Although statistical differences between groups existed, little practical difference exists.

The percentage of respondents in this sample who reported intention to call 9-1-1 in response to stroke signs or symptoms was lower than the percentage of BRFSS survey respondents who replied they would call 9-1-1 if they thought someone was having a heart attack or stroke (86%) (17). This finding implies that the awareness of a stroke as necessitating immediate treatment does not transfer to awareness of which signs or symptoms are associated with stroke and the need for emergency care. The study most comparable with ours in how questions were asked was the validation study of the Stroke Action Test (22). The population in our study showed greater intention to call 9-1-1 for four signs or symptoms than the population in the Stroke Action Test: inability to speak correctly (72% vs 47%), facial droop (64% vs 47%), trouble seeing (33% vs 12%), and sudden arm weakness (50% vs 44%). In our study, as in previous ones, sudden visual difficulty was the least recognized symptom of stroke (17,20,35).

A number of factors contribute to the delay in seeking emergency care in response to the signs or symptoms of a stroke (11). Consistent with previous studies, our study found that the respondents' age, sex, and history of hypertension were not related to intent to call 9-1-1 in response to stroke signs or symptoms (11). Personal or family history of hypertension was not associated with higher odds of intention to call 9-1-1. This is an important finding that can inform practice. Because hypertension is a major risk factor for stroke, this finding suggests missed opportunities by health care providers and public health professionals to inform people with hypertension about their risk for stroke.

Prior experience with having a heart attack or stroke or with a family member who had a heart attack or stroke was not associated with greater odds of intent to call 9-1-1 for a stroke sign or symptom. This finding is consistent with the overall findings from the literature review conducted by Moser and colleagues (11) on delay in seeking care, but it is inconsistent with other studies (20). Higher education was associated with intent to call 9-1-1, which is consistent with the review article by Moser and colleagues and a study based on BRFSS findings (23).

This is one of two studies that examined history of delaying care. The other study that examined history of delaying care did not find an association with the intent to call 9-1-1 for stroke symptoms (21). However, that study was conducted among a more diverse population. The difference in findings may be due to insurance coverage, acculturation, or other factors.

This study has several limitations. The response rate (36%) was low, and nonresponse may be associated with knowledge of stroke signs or symptoms and the intent to call 9-1-1. However, the response rate is within the range of response rates commonly achieved in telephone surveys conducted since 2000, and little evidence suggests that a higher response rate would significantly change the findings (36). The potential influence of cell phone use on the response rate is minimal. In 2004, an estimated 1.8% of older adults lived in cell-phone–only households and, in the Northeast, only 3.9% of households have cell phones only (37). Because the study population was primarily white and well educated, the findings are not generalizable to other race or ethnic populations or to a population that is less educated. Because the survey was conducted to evaluate a media campaign, more detailed questions about insurance coverage and knowledge of signs and symptoms were not asked. Respondents were not asked about facial drooping in themselves. This study did not measure actual use of 9-1-1. However, findings from studies on actual 9-1-1 use are difficult to interpret because of the wide range (34%–71%) of stroke patients who arrive by ambulance (7,12-15.)

Despite these limitations, this study has several strengths. It has a large sample size, which improves statistical power and generalizability. The use of unprompted questions designed to assess behavioral intention to call 9-1-1 in response to stroke signs or symptoms is arguably a more accurate reflection of the public's true intent to call 9-1-1. In most studies, when a respondent is presented with a symptom and asked whether it is a symptom of stroke (16,20,23), correct responses are higher than when a respondent is asked to list stroke symptoms (20). Previous research shows that much of the public understands that stroke needs emergency care (20) but that knowledge of stroke symptoms is low (23).

The multivariate modeling takes into account many potential confounders, including demographics and medical history. Perhaps one of the greatest strengths of this study is that it measured history of delay in getting medical care, which has rarely been evaluated in stroke awareness research. Because reasons for delaying seeking treatment for acute coronary syndrome and stroke share some similarities (11), findings from the coronary literature might be relevant to stroke. A delay in seeking immediate medical care may reflect increased use of self-care and problem-solving or reflect the use of defense mechanisms or emotional coping strategies (e.g., minimizing signs and symptoms, denial of serious illness, an unwillingness to trouble others, embarrassment to seek help) (38,39).

The American Heart Association council statement (11) notes that most research addressing the delay in stroke care has focused on decreasing in-hospital treatment delays (11), yet research has suggested that a large portion of the delay in receipt of care is due to prehospital delays (5,15). This study shows that many people do not interpret stroke signs or symptoms as urgent enough to call EMS. Understanding determinants of health care-seeking behavior is important for decreasing the proportion of people who delay seeking emergency care. More research is needed to understand these determinants. The findings from our study suggest that educational efforts should focus on increasing the perception of stroke symptoms as severe and the recognition of the need to call 9-1-1. Studies such as this one, which examined factors that contribute to awareness of the necessity to call 9-1-1, can inform educational campaigns targeting stroke-related behavior change.

## Figures and Tables

**Table 1:** Characteristics of Survey Sample (n = 1789), Four Counties, Upstate New York, July–September 2006

Characteristic	No.^a^ (%)
**Sex**	
Male	635 (35)
Female	1154 (65)
**Age, y**	
30–44	452 (25)
45–64	881 (49)
≥65	456 (25)
**Race/Ethnicity**	
White	1547 (88)
Black	99 (6)
Hispanic	60 (3)
Other	59 (3)
**Educational attainment**	
High school/GEDor less	564 (32)
College education or degree	858 (48)
Graduate education or degree	355 (20)
**Insurance coverage**	
No	80 (4)
Yes	1707 (96)
**Respondent or family member has or had hypertension**	
No	670 (38)
Yes	1114 (62)
**Respondent or family member had a heart attack or stroke**	
No	1119 (63)
Yes	664 (37)
**Respondent has delayed getting medical care in the past 6 months**	
No	1205 (68)
Yes	580 (32)

GED indicates General Educational Development certificate.

a Totals may not add up to 1789 because of missing data.

**Table 2:** Survey Respondents (%) Who Indicated Intention to Call 9-1-1 for Each Stroke Sign or Symptom, by Selected Characteristics^a^, Four Counties, Upstate New York, July–September 2006

Characteristic	Inability to Speak Correctly		Trouble Seeing or Double Vision		Facial Droop^b^		Arm Weakness	

	% (95% CI)	*P* Value	% (95% CI)	*P* Value	% (95% CI)	*P* Value	% (95% CI)	*P* Value
Overall	72.4 (69.9- 74.8)	–	33.3 (30.7- 36.0)	–	64.3 (61.5- 66.9)	–	49.8 (46.9- 52.6)	–
**Sex**								
Male	72.3 (68.2- 76.1)	.95	36.1 (31.9-40.6)	.05	64.5 (60.0-68.8)	.90	50.9 (46.3-55.5)	.45
Female	72.5 (69.4-75.4)		30.7 (27.7- 33.9)		64.0 (60.7- 67.2)		48.7 (45.4-52.1)	
**Age, y**								
<65	72.7 (69.8-75.5)	.56	33.2 (30.2- 36.3)	.81	66.2 (63.0-69.2)	.002	50.7 (47.5-53.9)	.14
≥65	71.1 (66.1-75.6)		33.8 (29.9-39.2)		56.4 (50.9-61.7)		45.9 (40.5-51.4)	
**Race/ethnicity**								
White	72.4 (69.7-74.9)	.59	32.5 (29.8-35.3)	.06	64.4 (61.5-67.2)	.86	49.9 (46.9-52.9)	.80
Other	74.6 (66.6-81.2)		41.0 (32.5- 50.1)		65.2 (56.3-73.2)		51.1 (42.3-59.9)	
**Educational attainment**								
High school/GED or less	70.5 (65.8-74.8)	.27	34.0 (29.4-39.0)	.50	59.9 (54.8-64.8)	.07	45.9 (30.9-51.0)	.08
College education or degree	72.2 (68.5-75.6)		31.9 (28.3- 35.7)		67.1 (63.2-70.8)		49.9 (45.8-53.9)	
Graduate education or degree	76.2 (69.9- 74.8)		35.9 (30.2-41.1)		63.5 (57.4-69.2)		55.2 (49.0-61.2)	
**Insurance coverage**								
Yes	72.7 (70.1-75.1)	.43	33.0 (30.4-35.7)	.53	64.0 (61.2-66.7)	.60	49.5 (46.6-52.3)	.56
No	68.0 (55.2-78.6)		37.2 (25.2-51.0)		67.4 (54.2-78.3)		53.5 (40.4-66.2)	
**Respondent or family member has or had hypertension**								
Yes	74.6 (71.4- 77.5)	.04	31.8 (28.6- 35.2)	.19	64.3 (59.6-68.3)	.93	51.1 (47.5-54.6)	.27
No	69.4 (65.1- 73.3)		35.4 (31.2-39.9)		64.1 (60.8-67.7)		47.8 (43.3-52.4)	
**Respondent or family member had a heart attack or stroke**								
Yes	74.2 (70.2- 77.9)	.29	29.0 (25.2- 33.2)	.01	63.6 (59.0-67.9)	.70	53.6 (49.1-58.1)	.04
No	71.5 (68.3- 74.6)		35.6 (32.3-39.1		64.6 (61.2-68.0)		47.6 (44.0-51.1)	
**Respondent delayed getting health care in the past 6 months**								
Yes	68.5 (63.8-72.8)	.02	28.7 (24.4-33.4)	.02	62.8 (57.9-67.5)	.44	47.4 (42.4-52.4)	.24
No	74.5 (71.5- 77.3)		35.6 (32.5-38.9)		65.1 (161.8-68.3)		51.0 (47.7-54.4)	

CI indicates confidence interval; GED, General Educational Development certificate.

a *P* values were determined by design-based *F* test.

b Asked about symptom in another person only.

**Table 3:** Association of Intention to Call 9-1-1 With Selected Survey Respondent Characteristics, by Stroke Sign or Symptom^a^, Four Counties, Upstate New York, July–September 2006

Characteristic	Inability to Speak Correctly	Trouble Seeing or Double Vision	Facial Droop^b^	Arm Weakness

	Adjusted Odds Ratio (95% CI)	Adjusted Odds Ratio (95% CI)	Adjusted Odds Ratio (95% CI)	Adjusted Odds Ratio (95% CI)
**Age, y**				
30-64	Ref	Ref	Ref	Ref
≥65	0.86 (0.64-1.16)	1.03 (0.77-1.38)	0.67 (0.51-0.89)	0.80 (0.61-1.05)
**Sex**				
Female	Ref	Ref	Ref	Ref
Male	0.98 (0.76-1.26)	1.24 (0.97-1.58)	0.99 (0.77-1.26)	1.04 (0.83-1.32)
**Race**				
Nonwhite	Ref	Ref	Ref	Ref
White	0.80 (0.52-1.22)	0.70 (0.45-1.00)	0.93 (0.62-1.40)	0.88 (0.60-1.29)
**Educational attainment**				
High school/GED or less	Ref	Ref	Ref	Ref
College education or degree	1.10 (0.82-1.46)	0.95 (0.72-1.26)	1.30 (0.99-1.72)	1.17 ( 0.89-1.53)
Graduate education or degree	1.39 (0.97-2.00)	1.13 (0.80-1.59)	1.12 (0.80-1.57)	1.51 (1.09-2.10)
**Insurance coverage**				
No	Ref	Ref	Ref	Ref
Yes	0.99 (0.55-1.78)	0.69 (0.38-1.26)	0.82 (0.45-1.49)	0.69 (0.39-1.23)
**Respondent or family member has or had hypertension**				
No	Ref	Ref	Ref	Ref
Yes	1.30 (0.99-1.72)	0.93 (0.71-1.21)	1.08 (0.83-1.40)	1.09 (0.85-1.40)
**Respondent or family member had a heart attack or stroke**				
No	Ref	Ref	Ref	Ref
Yes	1.10 (0.83-1.45)	0.75 (0.58-0.98)	0.96 (0.74-1.24)	1.27 (0.99-1.62)
**Respondent has delayed getting medical care in the past 6 months**				
No	Ref	Ref	Ref	Ref
Yes	0.76 (0.58-1.00)	0.69 (0.53-0.91)	0.85 (0.65-1.11)	0.80 (0.63-1.03)

CI indicates confidence interval; Ref, referent group; GED, General Educational Development certificate.

a The entries are adjusted odds ratios from a multiple logistic regression model for each symptom, weighted to reflect the study population.

b Asked about symptom in another person only.

## Appendix

### Survey items

1. What would you do if YOU experienced an inability to speak correctly? For instance, people you were talking with could not understand you, they said you were slurring or using inappropriate words. Would you . . .
2. What would you do if you saw or heard ANOTHER PERSON speak in a way that you could not understand? For instance, another person was slurring words or using inappropriate words. The person could not repeat a simple sentence. Would you . . .
3. What would you do if YOU experienced sudden trouble seeing or double vision? Would you . . .
4. What would you do if you heard ANOTHER PERSON complain about having sudden trouble seeing or double vision? Would you . . .
5. What would you do if you saw ANOTHER PERSON whose face looked uneven? It drooped on one side or they could not smile evenly. His or her smile didn't look like it usually does. Would you . . .
6. What would you do if YOU experienced sudden weakness in one arm? Would you . . .
7. What would you do if you heard ANOTHER PERSON say that he or she was suddenly feeling weak in one arm, or complain about a tingling sensation and numbness in the arm? Would you . . .

### Response options to survey items

1. Wait, watch symptoms, and then decide what to do
2. Call a family member or friend
3. Call a doctor or nurse
4. Call 9-1-1 or EMS [emergency medical services]
